# An exploration of the structure and understanding of *Xin* (“信”) in Chinese culture: the development of a theoretical model and questionnaire instruments

**DOI:** 10.3389/fpsyg.2026.1762903

**Published:** 2026-02-27

**Authors:** Lining Lin, Dian Jing, Changyu Sun

**Affiliations:** 1 Center for Zhouyi & Ancient Chinese Philosophy, Shandong University, Jinan, China; 2 Student Counseling Center, Shandong University, Jinan, China

**Keywords:** analytical psychology, Chinese culture, personality quality, psychological structure of *Xin*, *Xin* (“信”)

## Abstract

**Introduction:**

This study investigates the multifaceted construct of *Xin* (“信”) within Chinese culture, reconceptualizing it as a core personality quality that extends beyond mere honesty or trustworthiness.

**Methods:**

Employing a mixed-methods approach, we integrated grounded theory and quantitative scale development to articulate the structure of *Xin* and its psychological significance. Three sequential studies were conducted: (1) Through in-depth interviews with 22 Chinese participants, a five-dimensional model of Xin was constructed, comprising relational domains between humans and heaven and earth, humans and social culture, the self, individual archetypes, and connective integration. (2) A corresponding questionnaire was developed and validated via exploratory factor analysis (Sample 1, *N* = 231) and confirmatory factor analysis (Sample 2, *N* = 814), confirming the five-factor structure. (3) The questionnaire was administered to a broader sample (Sample 3, *N* = 807) to assess recognition and identification levels across these dimensions..

**Results:**

Study 1 established a five-dimensional model of *Xin*. Study 2 confirmed this structure via EFA and CFA, with good psychometric properties (α = .951–.954; CFI = .913, RMSEA = .051). Study 3 showed that *Xin* is perceived most strongly in relational connectivity and least in archetypal manifestations, with significant variations by gender, education, and ethnicity.

**Discussion:**

These findings illuminate *Xin* as a complex personality construct integral to Chinese cultural identity and personal development, offering insights for its cultivation in psychological practice and everyday life.

## 1 Introduction

Chinese characters possess a unique semantic richness, with each character often encompassing a spectrum of meanings. The character *Xin* (“信”) encompasses multiple dimensions, such as integrity, honesty, self-confidence, belief, faith, conviction, and a letter. Since ancient times, the Chinese have regarded *Xin*, or integrity, as an important criterion for distinguishing between sages, gentlemen, and villains, and have used it as a fundamental basis for evaluating individuals (Jiang, 2004); in Western traditions, shaped by the spirit of contract, integrity is closely associated with rules, sense of responsibility, and work commitment (Camara and Schneider, 1994). In modern society, integrity, as one of the dimensions in the HEXACO (honesty–humility, emotionality, extraversion, agreeableness, conscientiousness, and openness to experience) model of personality developed by Ashton and Lee (2008), is an important factor in building harmonious interpersonal relationships and fostering healthy economic and social development. However, there are few empirical studies on the concept of integrity in the field of psychology in China, and most have focused on a specific group. For example, in a value survey, Zhang (1998) argued that honesty is one of the values that college students are most willing to prioritize. Gui (2004) posited that *Cheng* (“诚”) encompasses honesty and the absence of deceit, while *Xin* comprises four dimensions: of trust, commitment, trustworthiness, and credit. Based on this conceptualization, she developed two questionnaires to assess these constructs among college students. The results revealed that college students assign varying degrees of importance to each dimension of integrity, in the following descending order of importance: credit, honesty, reliability, commitment, absence of deceit, and trust. Zhang and Wang (2003) explored interpersonal trust and interaction anxiety among 600 college students in the Nanchang area and found that gender and age have an impact on interpersonal trust relationships. Notably, in most studies, the research subjects were urban citizens. For example, Cheng (2002) conducted a questionnaire survey to examine the trust status among citizens of Jinan and found that women exhibited a higher degree of interpersonal trust than men, and older people demonstrated a higher degree of interpersonal trust than children. Li and Liang (2002) developed a trust questionnaire for Guangdong citizens, the empirical evidence indicating that the establishment of trust among Chinese people is affected by kinship, affection, gender, age, and cultural level.

In general, research on the construct of *Xin* within the Chinese psychological community has predominantly focused on the specific applications and social ramifications of related concepts such as integrity, trust, and credibility. To date, there has been limited effort to systematically delineate the conceptual underpinnings, scope, and structure of *Xin* that integrates these related notions. Moreover, the impact of *Xin* on individual personality and inner psyche, which could be significant, remains underexplored.

The level of social trust is directly linked to social culture (Fukuyama, 2001), and many scholars have explored the differences in *Xin* between Eastern and the Western cultures, as well as the underlying causes of these differences. For example, Wu and Huang (2010) and Yuan (2014) observed that the concept of integrity in foreign countries typically includes honesty, trustworthiness, and responsibility, while the Chinese perspective encompasses four factors: honesty, credibility, trust, and responsibility. According to Yang and Peng (1999), competence and responsibility of a trusted party are valued more in trust relationships in the West, while interpersonal factors such as consanguinity and friendship are emphasized in China. Considering the influence of different historical cultures and social styles on individual psychological states, it is crucial to approach psychological issues in China within the framework of Chinese culture, particularly through the lens of *Xin*.

*Xin* is a dynamic process evolving from establishment to development, breakdown, and repair – throughout this process, various factors affect an individual’s judgement of trust (Khodyakov, 2007). Therefore, solely exploring the structure of *Xin* and the factors affecting it through questionnaire measurements or experiments can lead to contradictory and one-sided results, as shown by Rotter (1967) and Wrightsman (1974), who examined the cognitive content or behavioral performance of honesty without considering socio-cultural environmental factors. Wu and Huang (2010) observed that there are individual differences and factors influencing research on integrity, such as limited rationality and personality traits. Therefore, it is more appropriate to adopt a multi-dimensional perspective, incorporating both qualitative and quantitative research.

Taking this context into account, the present study combined qualitative and quantitative research methods to investigate the structure and the meaning of *Xin* in Chinese culture. It drew upon on the philosophical concepts of the *I Ching*—an ancient Chinese divination text with a profound influence on Chinese philosophy—as well as principles from analytical psychology. Through interviews and grounded theory, we aimed to construct the *Xin* system structure within Chinese culture. Building upon the theoretical model of *Xin* and its structure within the context of Chinese culture, we then constructed a questionnaire designed to measure *Xin* and its structural dimensions. Finally, employing this newly developed questionnaire, we explored the extent to which Chinese individuals understand and identify with the concept of *Xin* within their cultural milieu.

This study integrates qualitative and quantitative methods to explore the structure and meaning of *Xin* within Chinese culture, drawing on classical philosophy (e.g., the *I Ching*) and analytical psychology. We aim to construct a theoretical model of *Xin*, develop a validated measurement tool, and examine how contemporary Chinese individuals perceive and identify with this fundamental personality quality. Partial data from the study involves informed consent and is temporarily not fully disclosed.

## 2 Study 1: qualitative research on *Xin* and its structure in Chinese culture

### 2.1 Research subjects

The aim of Study 1 was to qualitatively explore the inherent structure and personal significance of *Xin* within Chinese culture, specifically conceptualizing it as a multifaceted and stable personality trait. By examining how individuals perceive, experience, and embody *Xin* in their lives, we sought to construct a foundational model of its psychological architecture from an emic perspective.

### 2.2 Method

Grounded theory is a classical paradigm in qualitative research. It is a methodological approach through which researchers, without predefined theoretical assumptions, conduct gradual inductive analysis of firsthand observational data, form a three-tiered coding system, and ultimately develop theoretical constructs (Liu et al., 2019). In accordance with the recommendations of Lincoln and Guba (1985), who suggested that a sample size of more than 12 participants is necessary for in-depth interviews in qualitative research, the current study selected a total of 22 interviewees. This sample size is consistent with the requirements for qualitative research sample adequacy (Chen et al., 2016). The sample included 15 males and 7 females, with ages ranging from 26 to 65 years. Participants were drawn from diverse occupational backgrounds, including university professors, corporate employees, civil servants, self-employed business owners, psychological counselors, and retired professors.

### 2.3 Research instruments

A digital voice recorder and the qualitative research analysis software NVivo 14.0 (hereinafter referred to as N14) were utilized. N14 is a powerful tool designed to facilitate the indexing, searching, and theorizing of non-numerical and unstructured textual data (Wang et al., 2019). Specifically, it supports researchers in conducting tasks such as coding and searching, generating rules, creating indexes, establishing logical relationships, building conceptual networks, and constructing theoretical models. These capabilities are particularly relevant for elucidating the construct of *Xin* and its structure within the context of Chinese culture. During the data analysis process, we employed a continuous comparative approach, iteratively refining and revising the emerging theory until theoretical saturation was achieved.

### 2.4 Interview procedures

The design of the interview outline followed the principle that “interview questions should be as open-ended as possible for the interviewees” (Wang et al., 2021). That is, based on clear question settings, the interview aimed to maximize interviewees’ desire to express themselves. The final four interview questions were as follows: (1) When it comes to *Xin* in Chinese culture, what comes to mind? (2) How do you understand the concept of *Xin* in Chinese culture? (3) How do you think *Xin* affects your daily life? (4) If you compare *Xin* with “unity of knowledge and action,” “unity of psyche and body,” “unity of universe and man,” or “supreme sincerity,” “supreme goodness,” and other concepts, do you think they are related? How do you understand them?

The interviews were structured. During the interviews conducted by the researcher every effort was made to ensure that each interviewee was able to answer all four questions fully, providing as much detailed information as possible. The interviewer (i.e., the researcher) adhered to an interview time of 15–30 min, depending on the familiarity and interest of the interviewees in the topic. Owing to time and location constraints, the interview format was categorized into face-to-face and online interviews. Online interviews were conducted using WeChat voice and video calls, Zoom or Voov Meeting. Before starting the interview, the interviewer used a Xunfei recorder to record the interview with the consent of the interviewees. At the end of the interviews, the audio material was transcribed into text and saved in the researcher’s personal laptop folder for use as interview material for this study.

### 2.5 Coding procedures

This study followed a grounded theory research approach and used content analysis for data interpretation. After organizing and proofreading the transcripts and other materials, they were imported into N14 software in the form of electronic texts. Based on the degree of abstraction of the textual data, three rounds of coding were conducted to abstract and categorize the concepts and connotations of *Xin* in Chinese culture.

Supporting open coding was applied to the concept, connotations, and impact of *Xin* on life in Chinese culture. Each sentence was coded using keywords from the original materials, resulting in 21 codes and 64 reference points. This process was completed independently by a single researcher.

Axial coding was used to sort the internal connections of the open coding results and merge codes with similar meanings. Three researchers reviewed the original materials and discussed them establish category definitions. Five categories were identified from the 21 open codes: cosmic *Xin*, archetypal *Xin*, social and cultural *Xin*, self- *Xin*, and *Xin* of connection.

Selective coding consists of the iterative process of reading and reflecting on textual data by combining axial codes into a core category. Based on the coding results, a preliminary theoretical model of *Xin* and its structure in Chinese culture was developed.

### 2.6 Results and reliability and validity analysis

Table 1 shows that 21 codes with 64 reference points were obtained in the open coding stage. In the axial coding stage, the 21 codes were merged into 5 main categories according to their content and meaning, namely “cosmic *Xin*,” “*Xin* between humans and social culture,” “self-*Xin* of individuals,” “archetypal *Xin* of individuals,” and “*Xin* of connection.” In the selective coding stage, the five categories were integrated to establish the core category, *Xin* in Chinese culture and its structure.

**Table 1:** The coding results of *Xin* and its structure in Chinese culture.

Selective coding	Axial coding	Supporting open coding	References
*Xin* and its structure in Chinese culture	*Xin* between humans and social culture	The social evolution of *Xin*	4
		The modern transformation of *Xin*	3
		The practical function of *Xin*	6
		The interpersonal relationships of *Xin*	6
		Token of *Xin*	2
		Virtue of *Xin*	2
	Self-*Xin* of individuals	*Xin* and self—awareness	3
		*Xin* and self—manifestation	5
		*Xin* and self—stability	3
		*Xin* and self—development	5
		*Xin* and inner self	2
	Cosmic *Xin*	Authentic existence	3
		The union of heaven and earth	3
		Connecting heaven and earth	3
		Nourishing and inclusive	3
		Heartfelt influence and transformation	3
	Archetypal *Xin* of individuals	*Shen* (“神”) and *Xin*(“信”)	1
		The unconscious existence of *Xin*	3
		The transformation of the unconscious by *Xin*	1
	*Xin* of Connection	*Xin* between higher position and lower position	2
		*Xin* between inside and outside	1

Once the coding was completed, the researcher conducted a theoretical saturation test using uncoded interview transcripts,. The results indicated that no new theoretical nodes emerged when the uncoded new material was analyzed and coded, all the content could be covered by the existing five main categories, indicating a high degree of theoretical saturation. Simultaneously, the researchers evaluated the coding model using the expert evaluation method (Chen, 1999) which can be used to assess the validity of qualitative research. The researchers invited two experts to read and discuss the verbatim interviews and the coding results at each level. Finally, they concluded that the results of the qualitative study on *Xin* and its structure within Chinese culture were valid.

### 2.7 Discussion

Study 1, through a rigorous grounded theory approach, successfully delineated a complex, multi-dimensional psychological structure of *Xin* in the Chinese cultural context. The derived five-factor model—encompassing Cosmic *Xin* , *Xin* between humans and social culture, Self-*Xin*, Archetypal *Xin* , and the *Xin* of connection—moves beyond a fragmented understanding of *Xin* as mere behavioral honesty or situational trust. Instead, it presents a coherent framework that captures *Xin* as a pervasive and organizing principle within the individual’s inner world. A closer examination of the dimensions reveals characteristics that are highly suggestive of a stable personality system rather than a collection of isolated beliefs.

The construct of Cosmic *Xin* denotes the connection and interactivity between humans and all entities within the universe. This dimension provides a transcendent, meaning-making framework, reflecting an individual’s sense of connection, harmony, and faithful alignment with the Cosmic order and nature. It is a definite and authentic objective reality, reflecting the transcendent, foundational, future-oriented, inclusive, nurturing, and certain nature of the relationship between humans and heaven and earth, the universe, and nature. However, it is also primal, deeply embedded between humans and heaven and earth, to the extent that it may even operate unconsciously. The process of recovering Cosmic *Xin* is essentially a practice of aligning human actions with the natural order, a process of “harmonizing humans with heaven.”

The *Xin* between humans and social culture refers to the connection of *Xin* between humans and social culture, which is an uncertainty. Social-Cultural *Xin* governs its expression in relational contexts. In the sociocultural context, *Xin* serves as an intermediate link in human social interaction (Liu et al., 2025). This dimension captures the interpersonal and normative facet of *Xin* as a personality trait. It involves the inclination to adhere to social norms of honesty, fulfill roles and commitments, and engage in trustworthy behavior within societal structures.

The dimension of self-*Xin* of individuals refers to the intrinsic *Xin* that exists within a person’s inner world. Self-*Xin* forms the stable, internal anchor of the personality. The dimension focus on self-awareness, self-consistency, and intrinsic authenticity, aligns closely with the core of personality, representing the internal, stable anchor of one’s identity.

The Archetypal *Xin* of individuals represents an eternal and unchanging form of *Xin* that has persisted from ancient times to the present. This dimension points to the deepest, often unconscious, layer of *Xin* within the personality structure. The conception of archetypal rooted in Jungian theory (Jung, 1968), the Archetypal *Xin* represents the profound, motivational underpinnings of individuals. It refers to innate, universal patterns related to trust, faith, and the potential for wholeness, which guide the individual’s psychological development. It serves as the wellspring from which heartfelt influence and transformation arise, converting the potential for life into life itself, and evolving individuals from an incomplete self to a complete and whole Self.

The dimension of *Xin* of connection signifies the role that *Xin* plays in integrating the individual’s inner world with the external environment, and bridging the relationship between heaven and earth and the individual self. This is the dynamic, integrative force that ensures coherence across these layers, making *Xin* a unified and operative trait. Specifically, *Xin* serves as a crucial link between an individual’s moral knowledge and moral practice, enabling the manifestation of inner goodness in external actions.

**Figure 1:**
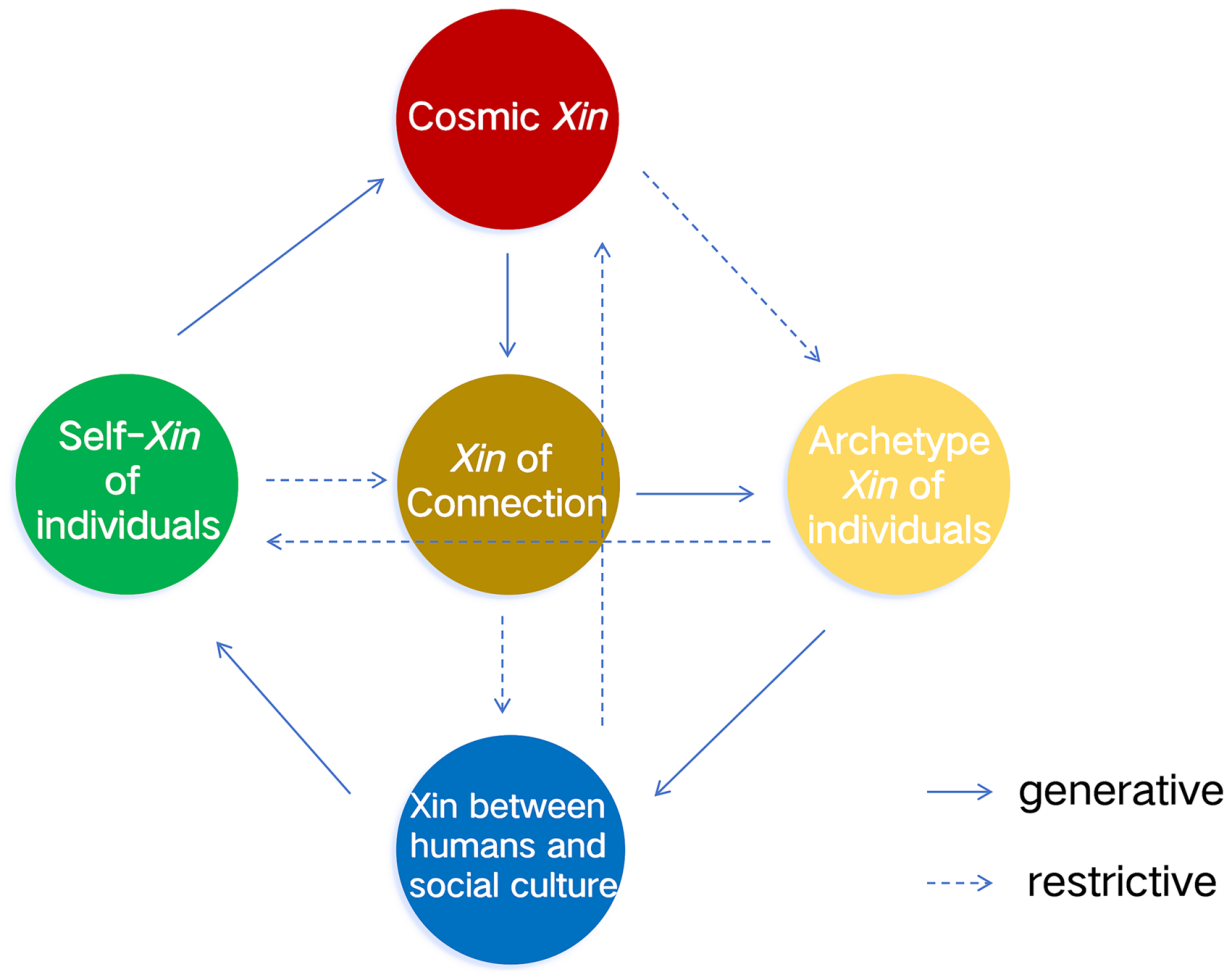
The model of *Xin* and its structure in Chinese culture.

The resonance of this five-factor structure with traditional Chinese frameworks, particularly the Five Elements theory, is not merely symbolic but theoretically significant. Figure 1 visualizes the five core dimensions of *Xin* conceptualized within a Chinese cultural framework, mapped onto the *Wu Xing* (五行and Five Elements) system. In traditional Chinese thought, the Five Elements have been correlated with the Five Virtues and concepts from the study of the *I Ching*. Scholars often map the four hexagram statements of *Qian* (“乾”): “Yuan, Heng, Li, Zhen (元亨利贞)”, onto the elements of Wood(“木”), Fire(“火”), Metal(“金”), and Water(“水”), which in turn represent spring, summer, autumn, and winter. These symbolize the four stages of development: initiation, flourishing, benefit, and steadfastness, embodying the moral connotations of benevolence (“仁”), propriety (“礼”), righteousness (“义”), and wisdom (“智”). Specifically, *Yuan*(“元”) corresponds to the benevolence of spring, *Heng*(“亨”) to the propriety of summer, *Li*(“利”) to the righteousness of Metal (autumn), and *Zhen*(“贞”) to the wisdom of winter (Water). Earth(“土”), possessing the capacity for transformation and generation, serves as the precondition and dynamic force enabling the transition among the four seasons, and is thus distributed throughout them (Li, 2016).

The connections within the five-dimensional model correspond accurately to the theoretical mappings and conceptual meanings outlined above. The model illustrates the dynamic, systemic nature of *Xin* as a personality construct: Wood corresponds to Self- *Xin*; Fire corresponds to Cosmic *Xin*; Earth corresponds to *Xin* of connection; Metal corresponds to Archetypal *Xin* of individuals; and Water corresponds to *Xin* between human and social cultural. It should be noted that this correspondence represents a preliminary and speculative alignment drawn from cultural theory, and has not been subjected to systematic empirical validation. Although the model is just a hypothesis, it has not yet undergone rigorous verification. The interrelationships among these dimensions, governed by the generative (相生) and restrictive (相克) cycles of the Five Elements, form an integrated psychological system that maintains dynamic equilibrium and facilitates personal development.

This congruence suggests that the identified structure of *Xin* is not an arbitrary construct but is deeply embedded within the indigenous cultural worldview. It reflects a culturally salient way of organizing fundamental aspects of human experience and disposition. The fact that an empirically derived psychological model aligns with a foundational cultural paradigm greatly strengthens the ecological validity of the construct and bolsters the argument that we are mapping a central aspect of “personhood” as conceived within Chinese culture.

In conclusion, the qualitative findings of Study 1 provide a rich, nuanced, and theoretically grounded model of the psychological structure of *Xin*. While it stops short of definitive empirical validation as a personality trait, the nature of the dimensions—spanning from transcendent relationality to deep intra-psychic processes—lends compelling qualitative support to the thesis that *Xin* embodies the complexity, stability, and organizational function characteristic of a core personality construct. This model, therefore, serves as an indispensable theoretical foundation. It not only captures the profound cultural connotations of *Xin* but also explicitly frames it as a viable target for personality psychology research, thereby paving the way for the quantitative operationalization and hypothesis testing undertaken in Study 2.

## 3 Study 2: the reliability and validity test of the questionnaire on *Xin* in Chinese culture and its structure

### 3.1 The participants

Participants in this study were aged 18 years or older and residing in mainland China. The sample was divided into two cohorts. For Sample One, a total of 260 questionnaires were administered, with 29 excluded due to incomplete or invalid responses, resulting in 231 valid responses (response rate = 88.8%). The gender distribution of Sample 1 included 102 males (44.16%) and 129 females (55.84%). For Sample Two, 862 questionnaires were distributed, with 48 excluded for similar reasons, yielding 814 valid responses (response rate = 94.4%). Sample Two included 362 male (44.47%) and 452 (55.53%) female participants.

### 3.2 Measurement instrument

The main measurement instrument of this study was a questionnaire on *Xin* in Chinese culture and its structure. The initial items of the questionnaire were developed using the following steps:

Step 1: A researcher and a professor from a university’s psychological counseling center (Ph.D. in Applied Psychology) independently compiled items based on the structure, dimensions, and keywords obtained from Study 1, resulting in a total of 75 items.

Step 2: The researcher invited graduate psychology students (three master’s and five doctoral students) and non-psychology majors (two students) to discuss the questionnaire items. Using clarity of meaning and expression as the criteria, they discussed, modified, and preliminarily screened the 75 items.

Step 3: Three psychology professors were invited to conduct expert reviews of the questionnaire’s dimensions and items. They rated the relevance (or representativeness) of each item to the corresponding content dimension on a 4-point scale (1 for “irrelevant,” 2 for “weakly relevant,” 3 for “moderately relevant,” and 4 for “very relevant”). Items scoring 1 or 2 were deleted, those scoring 3 were considered for retention, and those scoring 4 were retained.

A total of 52 items were selected for the initial questionnaire. The questionnaire used a Likert 5-point scale, where 1 means “strongly disagree,” 2 means “somewhat disagree,” 3 means “do not know,” 4 means “somewhat agree,” and 5 means “strongly agree” (Zhao et al., 2017). Higher scores indicated a greater degree of the individual’s understanding and identification of *Xin* within Chinese culture.

### 3.3 Method

To ensure the data collected from Sample One were suitable for factor analysis, an initial suitability test was conducted. Subsequently, an exploratory factor analysis (EFA) was performed using principal component analysis on the data from Sample One. The EFA was initially conducted on 47 items. The sample data were analyzed using the maximum likelihood estimation method with AMOS 26.0. Based on model fit criteria established in prior research, items with factor loadings less than 0.5 were removed, specifically items 22, 32, and 38. This resulted in all remaining factor loadings being greater than 0.6, indicating a satisfactory standard. Additionally, items with multiple excessively high modification indices (MI) in the regression analysis of the same factor were also deleted, namely, items 21 and 28, leaving a total of 42 items. Confirmatory factor analysis (CFA) was then conducted on the 42 items. Finally, the reliability of the questionnaire was assessed using Cronbach’s *α* coefficient.

### 3.4 Results

#### 3.4.1 Discrimination

SPSS (version 26.0) software was used to conduct a preliminary analysis on the data of Sample One, calculating the critical ratio (CR value) to perform item analysis on the questionnaire of *Xin* in Chinese culture and its structure. By summing all the analysis items and dividing the data into high- and low-scoring groups based on the 27th and 73rd percentiles, an independent-sample *t* test was conducted on the two groups of samples and their differences were compared. A significant difference was observed in the scores between Sample One and Sample Two (*p* = 0.000), indicating good discrimination.

#### 3.4.2 Exploratory factor analysis

##### 3.4.2.1 Suitability test

The Kaiser–Meyer–Olkin value was 0.935 (>0.8), and the *χ*^2^ value of Bartlett’s sphericity test was 8175.142, with *df* = 1,326 and *p* = 0.000 (<0.001), indicating that the data were suitable for factor analysis.

##### 3.4.2.2 Principal component analysis

The results showed eight factors with eigenvalues > 1, among which the 6th, 7th, and 8th factors each contained one item. After applying different permutations and combinations and gradually removing items with factor loadings less than 0.7, Items 8, 20, 29, 34, and 44 were eliminated, leaving 47 items.

The results showed that there were five factors with eigenvalues > 1, and the cumulative variance explained by these factors was 66.07% (Table 2). Based on the results of the EFA and the dimensions to which each item was assigned, it was confirmed that the questionnaire on *Xin* in Chinese culture includes the five dimensions determined in the qualitative research: Cosmic *Xin*, *Xin* between humans and social culture, an individual’s self-*Xin*, an individual’s archetypal *Xin*, and *Xin* of connection. The catalogue of each item and the dimension coding are listed in Table 3.

**Table 2:** The structural factor characteristics, eigenvalues, and cumulative contribution rates of *Xin* in Chinese culture (*N* = 231).

Factor names	Eigen values	Cumulative contribution rate %
Cosmic *Xin*	16.386	34.864
*Xin* between humans and social culture	6.023	47.679
Self-*Xin* of individuals	3.968	56.122
Archetypal *Xin* of individuals	2.675	61.813
*Xin* of connection	2.000	66.070

**Table 3:** The structural matrix of *Xin* in Chinese culture after rotation (*N* = 231).

Items	Self-*Xin* of individuals	*Xin* between humans and social culture	Cosmic *Xin*	Archetypal *Xin* of individuals	*Xin* of connection
Q1	—	0.776	—	—	—
Q2	—	0.743	—	—	—
Q3	—	0.787	—	—	—
Q4	—	—	0.773	—	—
Q5	—	—	0.736	—	—
Q6	—	0.766	—	—	—
Q7	—	—	0.779	—	—
Q9	0.775	—	—	—	—
Q10	0.784	—	—	—	—
Q11	0.753	—	—	—	—
Q12	—	0.746	—	—	—
Q13	0.770	—	—	—	—
Q14	0.799	—	—	—	—
Q15	—	0.794	—	—	—
Q16	—	0.782	—	—	—
Q17	—	0.744	—	—	—
Q18	—	0.822	—	—	—
Q19	—	0.754	—	—	—
Q21	0.711	—	—	—	—
Q22	—	0.806	—	—	—
Q23	—	—	0.791	—	—
Q24	—	0.768	—	—	—
Q25	—	—	0.745	—	—
Q26	—	0.783	—	—	—
Q27	0.779	—	—	—	—
Q28	0.784	—	—	—	—
Q30	—	—	0.778	—	—
Q31	—	—	0.755	—	—
Q32	—	—	0.757	—	—
Q33	0.754	—	—	—	—
Q35	0.731	—	—	—	—
Q36	0.731	—	—	—	—
Q37	—	—	—	0.765	—
Q38	—	—	—	0.778	—
Q39	—	—	0.736	—	—
Q40	—	—	—	0.801	—
Q41	—	—	—	0.749	—
Q42	—	—	—	0.762	—
Q43	0.763	—	—	—	—
Q45	—	—	—	0.712	—
Q46	—	—	—	—	0.687
Q47	—	—	—	—	0.768
Q48	0.755	—	—	—	—
Q49	—	—	—	—	0.711
Q50	—	—	—	—	0.724
Q51	—	—	—	—	0.739
Q52	—	—	0.763	—	—

Extraction method: Principal component analysis. Rotation method: Kaiser normalized varimax rotation. Rotation converged in 6 iterations.

#### 3.4.3 Confirmatory factor analysis

**Figure 2:**
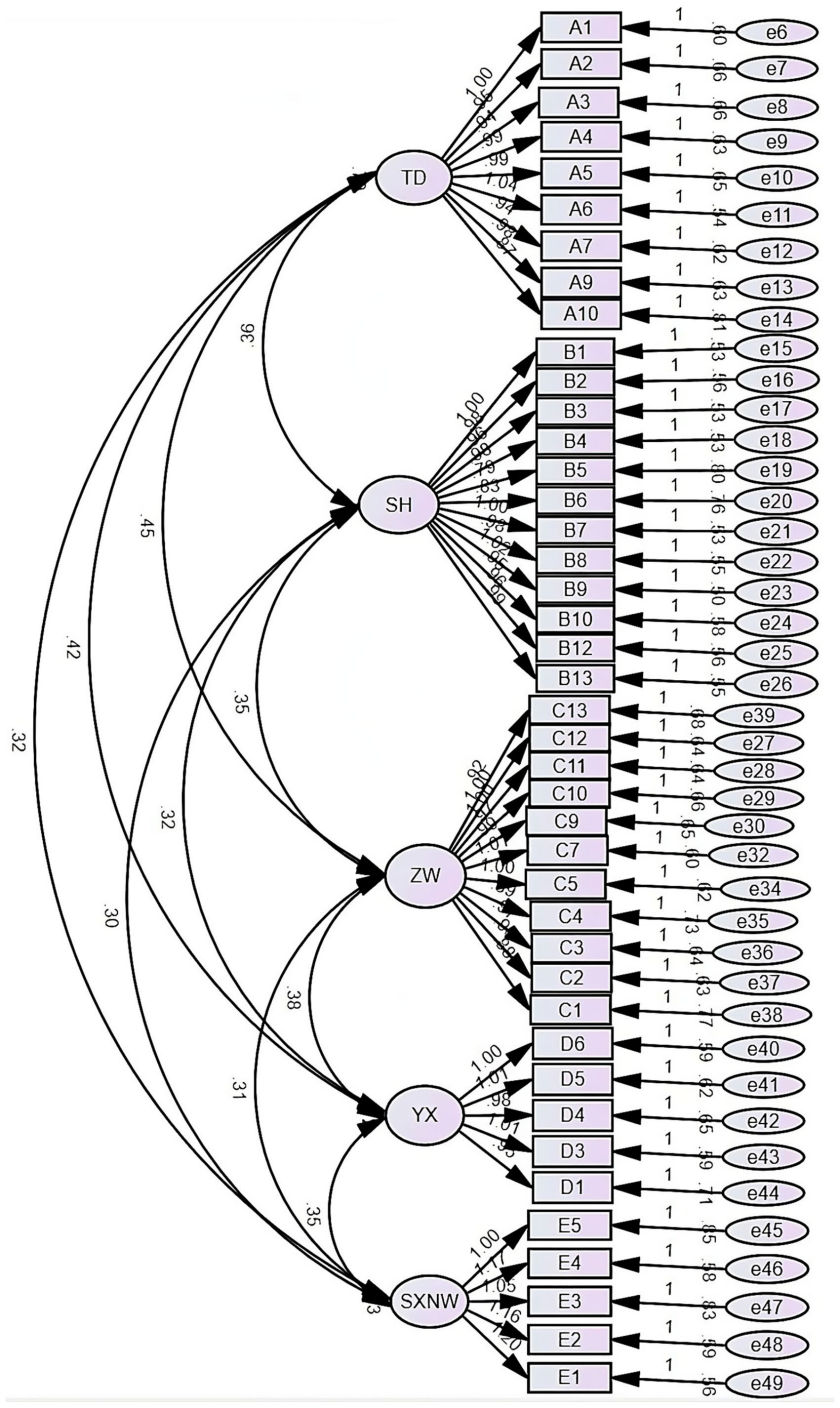
The validation path diagram of *Xin* and its structure in Chinese culture.

The results confirm that in the 5-factor model of *Xin* and its structure in Chinese culture (Figure 2), the comparative fit index (CFI = 0.913), normed fit index (NFI = 0.876), Tucker–Lewis index (TLI = 0.907), incremental fit index (IFI = 0.913), and goodness of fit index (GFI = 0.807) were all > 0.8. The root mean square error of approximation (RMSEA = 0.051 < 0.08), and the value of *χ*^2^/*df* was 3.079, indicating that the model fit was good (Table 4).

**Table 4:** Confirmatory factor analysis results of *Xin* in Chinese culture (*N* = 814).

Fit indices	*Χ*^2^/*df*	CFI	NFI	TLI	IFI	GFI	RMSEA
Fit values	3.079	0.913	0.876	0.907	0.913	0.807	0.051

#### 3.4.4 Internal consistency reliability

The results show that in Sample One (*N* = 231) and Sample Two (*N* = 814), the internal consistency coefficients of the questionnaire were 0.951 and 0.954, respectively, both exceeding 0.8. This indicates that the questionnaire on *Xin* in Chinese culture had good reliability and stability.

### 3.5 Discussion

The primary aim of study 2 was to create a reliable and valid measurement tool. The high internal consistency and good model fit indices confirm that the questionnaire meets standard psychometric requirements. Crucially, the instrument’s demonstrated ability to reliably measure variations across the five dimensions provides the necessary means to treat *Xin* as a measurable psychological construct that exhibits key properties of a personality trait—namely, individual differences and temporal stability. The questionnaire does not merely assess transient attitudes but captures a more enduring personal quality, as evidenced by the coherent and stable structure that persists across different samples. Therefore, while *Xin* can be consciously cultivated, its core, as measured, appears to function as a relatively stable lens through which individuals perceive and engage with the world, others, and themselves.

The empirical emergence of a stable five-factor structure is not only a psychometric achievement but also a finding of profound theoretical relevance. It resonates significantly with the philosophical importance of the number *five* in the *I Ching* philosophy (Richard, 1977), which symbolizes the Five Elements and represents a fundamental, holistic structure of the universe within Chinese cosmology. This congruence is not coincidental; it suggests that the measured psychological structure of *Xin* is deeply embedded within the Chinese cultural worldview. This alignment enhances the ecological validity of the model and strongly implies that we are capturing a culturally salient and meaningful organization of individual psychology. The fact that this empirical structure mirrors a core cultural paradigm lends considerable weight to the argument that *Xin* represents a central, culturally-grounded psychological characteristic with the potential to function as a stable personality disposition.

The questionnaire met the psychometric requirements, making it a reliable and valid measurement tool. The higher the score for each factor, the greater the understanding and recognition of the corresponding factor dimension. The degree to which the *Xin*’s personality level leans towards this dimension is also higher. In summary, this study provides researchers with a scientifically sound instrument to quantify individual differences in the psychological construct of *Xin*. The demonstrated reliability and validity of the measure are paramount. They lay a crucial foundation for future research to systematically investigate *Xin*’s nomological network—specifically, its relationship with established personality models (e.g., the Big Five), its predictive power over behavior and well-being, and its development across the lifespan. By establishing a robust measurement model, this research effectively bridges a profound cultural concept with the empirical field of individual differences, opening the door for rigorous inquiry into *Xin* as a pivotal personality quality within Chinese culture.

## 4 Study 3: understanding and analysis of the concept of *Xin* in Chinese culture

### 4.1 Research purpose

Study 3 used the questionnaire on *Xin* in Chinese culture and its structure to explore the current understanding and perception of *Xin* among Chinese people, focusing on its connotations and psychological significance. It also seeks to offer insights, directions, and inspiration for the practical application of *Xin* in psychological analysis.

### 4.2 Research design

#### 4.2.1 Research subjects

The research subjects were adults over 18 years old living within the territory of China.

#### 4.2.2 Research hypotheses

*Hypothesis 1*: The degree of recognition of Xin in Chinese culture is generally slightly above average.

*Hypothesis 2*: Gender has a significant impact on the recognition of *Xin* in Chinese culture.

*Hypothesis 3*: The degree of recognition of *Xin* in Chinese culture increases with age.

*Hypothesis 4*: Recognition of *Xin* in Chinese culture increases with educational level among Chinese people.

*Hypothesis 5*: Ethnicity has a significant impact on the recognition of *Xin* in Chinese culture.

*Hypothesis 6*: Chinese people’s recognition of *Xin* in interpersonal and social contexts, as well as *Xin* within the individual self, is significantly higher than in the other three dimensions.

*Hypothesis 7*: The Chinese people’s recognition of *Xin* in the relationship between humans and the universe, as well as *Xin* in individual archetypes, is significantly lower than in the other three dimensions.

#### 4.2.3 Statistical analysis

The collected data were analyzed using descriptive statistical statistics, a *t* test, and one-way analysis of variance with SPSS (version 26.0) software.

### 4.3 Participants

This study conducted a questionnaire survey among adults aged 18 years and older in China. A total of 860 questionnaires were distributed by random sampling, with 807 valid responses corresponding to an effective response rate of 93.8%. Questionnaires were sent to individuals from different provinces and cities, ethnic groups, age groups, educational levels, and occupations across the country to ensure a diverse and representative sample. The final data results show that, except for Taiwan and the Tibet Autonomous Region, the data cover 32 provinces nationwide.

Of the participants, 548 (67.9%) were female and 259 (32.1%) were male. Age distribution was as follows: 189 participants aged 18–25 years (representing 23.4% of the sample), 111 aged 26–30 years (13.8%), 188 aged 31–40 (23.3%), 208 were aged 41–50 (25.8%), and 111 aged over 50 years (13.8%). Regarding ethnicity, 727 participants were Han Chinese (90.1%) and 80 were from other ethnic minorities (9.9%). In terms of educational level, 50 participants had a high school diploma or lower (6.2%); 98 had an associate degree (12.1%), 414 had a bachelor’s degree (51.3%), and 245 had a master’s degree or higher (30.4%).

### 4.4 Method

In accordance with the research hypothesis, descriptive statistical analysis, t-test analysis, and one-way analysis of variance were conducted on the 807 valid data collected using the SPSS (version 26.0) software.

### 4.5 Results

#### 4.5.1 The overall degree of Chinese people’s recognition of *Xin* in Chinese culture

The analysis of the degree of participant recognition of *Xin* in Chinese culture and its structure, along with descriptive statistics on the five dimensions (Table 5), shows that the average score of Chinese people’s recognition of *Xin* in Chinese culture is 4.091. This value is moderately high, thereby verifying Hypothesis 1. Specifically, the average scores were as follows: 4.090 for the dimension of *Xin* between humans and the heaven and earth, 4.122 for *Xin* in social relationships, 4.093 for individual self-*Xin*, 3.957 for *Xin* in individual archetypes, and 4.183 for *Xin* in connections.

**Table 5:** Descriptive statistical results (*N* = 807).

Dimension	Samples	Minimum value	Maximum value	Mean	Standard deviation
Cosmic *Xin*	807	1.00	5.00	4.0896	0.60152
*Xin* between humans and social culture	807	1.00	5.00	4.1220	0.53093
Self-*Xin* of individuals	807	1.09	5.00	4.0927	0.58468
Archetypal *Xin* of individuals	807	1.00	5.00	3.9566	0.68566
*Xin* of connection	807	1.00	5.00	4.1834	0.60278
Total score	807	1.10	5.00	4.0907	0.53595
Number of valid cases (in columns)	807	—	—	—	—

The results (Table 6) reveal that the degree of recognition of *Xin* of connection is the highest, followed by *Xin* between humans and social culture. There was no significant difference between the self-*Xin* of individuals and Cosmic *Xin* dimensions; furthermore, both types of *Xin* were lower than *Xin* between humans and social culture. The degree of recognition of the archetypal *Xin* of individuals was the lowest. Therefore, Hypotheses 6 and 7 were partially verified.

**Table 6:** Results of paired sample *t*-test (*N* = 807).

Dimension	*t*	*df*	Sig. (2-tailed)
Cos-SC	−2.623	806	0.009
Cos-S	−0.288	806	0.774
Cos-A	8.222	806	0.000
Cos-C	−6.024	806	0.000
SC-S	2.370	806	5.00
SC-A	9.321	806	0.018
SC-C	−3.612	806	0.000
S-A	8.984	806	0.000
S-C	−6.065	806	0.000
A-C	−12.985	806	0.000

Cos, Cosmic *Xin*; SC, *Xin* between humans and social culture; S, Self-*Xin* of individuals; A: Archetypal *Xin* of individuals; C, *Xin* of connection.

#### 4.5.2 Differences in the degree of recognition of Xin in Chinese culture among different groups

##### 4.5.2.1 Differences by gender

A marginally significant difference (*p* = 0.051) in the recognition of *Xin* in Chinese culture was observed (Table 7), with males having a lower degree of recognition than females. Significant gender-based differences were found in the dimensions of *Xin* between humans and the universe and the individual self-*Xin*. No significant differences were found in the dimensions of *Xin* related to social relationships and individual archetypes. A marginally significant difference (*p* = 0.052) was found for *Xin* in connections. Thus, Hypothesis 2 was partially verified.

**Table 7:** Results of independent samples *t*-test by gender (*N* = 807).

Dimension	Gender	Samples	Average value	*t*	Sig. (2- tailed)
Cosmic *Xin*	Male	259	4.0279	−2.009	0.045
	Female	548	4.1188		
*Xin* between humans and social culture	Male	259	4.0885	−1.232	0.218
	Female	548	4.1378		
Self-*Xin* of individuals	Male	259	4.0158	−2.578	0.010
	Female	548	4.1291		
Archetypal *Xin* of individuals	Male	259	3.9282	−0.810	0.418
	Female	548	3.9701		
*Xin* of connection	Male	259	4.1236	−1.942	0.052
	Female	548	4.2117		
Total scores	Male	259	4.0372	−1.952	0.051
	Female	548	4.1159		

##### 4.5.2.2 Differences by age

There was no significant difference in the recognition of *Xin* in Chinese culture and its dimensions among different age groups. Thus, Hypothesis 3 was not verified.

##### 4.5.2.3 Differences by educational level

There was a marginally significant difference (*p* = 0.063) in the recognition of *Xin* in Chinese culture according to educational level. Specifically, as educational level increased, participants tended to have a lower degree of recognition of *Xin* in Chinese culture. Among the dimensions, there were no significant differences in Cosmic *Xin*, *Xin* in social relationships and *Xin of connection* (*p* = 0.052). However, significant differences were found in individual self-*Xin* and *Xin* in individual archetypes. Thus, Hypothesis 4 was partially verified (see Tables 8, 9).

**Table 8:** Results of homogeneity of variance test (*N* = 807).

Dimension	Levene	df1	df2	Sig.
Cosmic *Xin*	0.169	3	803	0.917
*Xin* between humans and social culture	0.225	3	803	0.879
Self-*Xin* of individuals	0.055	3	803	0.983
Archetypal *Xin* of individuals	0.100	3	803	0.960
*Xin* of connection	0.215	3	803	0.886
Total scores	0.470	3	803	0.703

**Table 9:** Results of one-way ANOVA on educational level (*N* = 807).

Dimension	Educational level	Samples	Average value	*F*	Sig.
Cosmic *Xin*	High school and below	50	4.1511	1.081	0.356
	Associate degree	98	4.1020		
	Bachelor’s degree	414	4.1122		
	Master’s and above	245	4.0340		
*Xin* between humans and social culture	High school and below	50	4.1300	1.723	0.161
	Associate degree	98	4.1522		
	Bachelor’s degree	414	4.1516		
	Master’s and above	245	4.0582		
Self-*Xin* of individuals	High school and below	50	4.1745	2.791	0.040
	Associate degree	98	4.1280		
	Bachelor’s degree	414	4.1267		
	Master’s and above	245	4.0045		
Archetypal *Xin* of individuals	High school and below	50	4.1880	4.929	0.002
	Associate degree	98	4.0082		
	Bachelor’s degree	414	3.9874		
	Master’s and above	245	3.8367		
*Xin* of connection	High school and below	50	4.2240	1.274	0.282
	Associate degree	98	4.2102		
	Bachelor’s degree	414	4.2092		
	Master’s and above	245	4.1208		
Total scores	High school and below	50	4.1600	2.445	0.063
	Associate degree	98	4.1222		
	Bachelor’s degree	414	4.1197		
	Master’s and above	245	4.0148		

##### 4.5.2.4 Ethnic differences

No significant differences were observed among the different ethnic groups (Table 10). However, significant differences were found in individual self-*Xin* and Xin in individual archetypes, with ethnic minorities scoring significantly higher than the Han ethnic group in these areas. Thus, Hypothesis 5 was partially verified.

**Table 10:** Results of independent samples *t*-test by ethnicity (*N* = 807).

Dimension	Ethnicity	Samples	Average value	Mean Difference	*t*	Sig. (2-diled)
Cosmic *Xin*	Han ethnic group	727	4.0816	−0.07875	−1.263	0.207
	Others	79	4.1716			
*Xin* between humans and social culture	Han ethnic group	727	4.1133	−0.09093	−1.453	0.147
	Others	79	4.2046			
Self-*Xin* of individuals	Han ethnic group	727	4.0773	−0.04929	−2.346	0.019
	Others	79	4.2394			
Archetypal *Xin* of individuals	Han ethnic group	727	3.9392	−0.11327	−2.186	0.029
	Others	79	4.1165			
*Xin* of connection	Han ethnic group	727	4.1785	−0.04189	−0.725	0.468
	Others	79	4.2304			
Total scores	Han ethnic group	727	4.0798	−0.08813	−1.808	0.071
	Others	79	4.1945			

### 4.6 Discussion

The application of the newly developed questionnaire in Study 3 provides a preliminary mapping of how the psychological structure of *Xin* is understood and embodied within a contemporary Chinese sample. The moderately high overall level of recognition (M = 4.09) suggests that *Xin*, as a multifaceted personal quality, retains significant relevance in the modern Chinese psyche. These results create a notable paradox: while explicitly endorsed as a valued trait at the conceptual level, its behavioral manifestation is weakened in everyday practice (Zhang, 2016). One participant’s poignant remark—“It is not that *Xin* itself lacks importance; rather, in today’s society, no one pays it any heed”—reflects a social reality where this foundational personality quality has been culturally marginalized into a kind of “dormant virtue.”

More revealing from a personality assessment perspective is the observed response pattern during measurement. Some participants reported difficulty grasping items pertaining to deeper dimensions of *Xin*. Notably, these participants displayed a marked “acquiescence bias,” systematically selecting “agree” to “strongly agree” options. This measurement artifact suggests a psychologically significant phenomenon: when navigating trait-relevant judgments, individuals’ self-perception appears susceptible to social desirability, complicating authentic trait expression.

The resulting “pseudo-faith”—a temporary psychological alignment constructed through self-persuasion—may function as a defense mechanism, which aligns both internally and externally. While serving short-term adaptive purposes, this phenomenon ultimately signifies a rupture between trait cognition and trait-consistent behavior, revealing a structural fragility in the personality system itself. These observed fragilities find further elaboration in the systematic patterns of differential recognition across *Xin*’s dimensions and demographic groups. The significant variations in how different aspects of this personality disposition are recognized offer profound insights into the complex interplay between its psychological structure and the contemporary socio-cultural environment.

#### 4.6.1 The hierarchical recognition of dimensions and its personality implications

The finding that *Xin* of connection received the highest recognition, while Archetypal *Xin* of individuals was the lowest, is particularly revealing. The high recognition of connective *Xin* underscores a culturally prioritized aspect of this personality quality: the importance of maintaining harmony and consistency between one’s inner world and outer actions, and between the self and the social or natural environment. *Xin* in connection refers to the connection of *Xin* between the inner self and the outside world, as well as between heaven and earth and individuals. It is intangible and formless, existing in the Cosmic *Xin*, *Xin* between humans and social culture, the self-*Xin* of individuals, and the archetype-*Xin* of individuals.

This can be interpreted as the social-behavioral expression of the *Xin* trait, which is most visible and actively reinforced in a collectivist culture (Jesse et al., 2013). Accordingly, individuals must find and achieve a balance and connection between the inner self and the external world as well as between nature and the self. Even “dis-*Xin*,” which includes acts such as deception and concealment, carries the meaning of connection.

Conversely, the lowest recognition of Archetypal *Xin* highlights a potential atrophy in the depth-psychological foundation of this personality system. This dimension, associated with the unconscious, the sacred, and the innate potential for wholeness, represents the most profound and stable core of the trait (Roesler, 2012). Its marginalization suggests that modern, fast-paced societal pressures, a focus on material success, and a lack of cultural discourse on these deeper layers may be hindering individuals from accessing and identifying with this fundamental aspect of their psychological makeup. This disconnection between the highly valued external congruence (Connection) and the under-identified internal foundation (Archetype) may point to a source of internal conflict and the “pervasive sense of distrust” noted in society, reflecting a personality structure under stress.

#### 4.6.2 Demographic variations as a lens into trait formation and expression

The demographic differences further illuminate how the development and expression of this personality quality are shaped by socio-cultural factors.

##### 4.6.2.1 Gender differences

The generally higher recognition among females, particularly in Self-*Xin* of individuals and the *Xin* of connection, aligns with social role theory and cultural expectations. The traditional association of femininity with the *Kun* (“坤”) hexagram (The Receptive), which embodies nurturing, integration, and inner stability, may socialize women to be more attuned to their internal states and the relational harmony that connective *Xin* facilitates. This suggests that cultural archetypes and gender roles can differentially sculpt the manifestation of a shared personality trait.

##### 4.6.2.2 Educational level differences

The negative correlation between education level and recognition of Self-*Xin* and Archetypal *Xin* is a critical finding. It suggests that advanced education, while fostering rationality and abstract thinking, may inadvertently lead individuals to prioritize external, logical frameworks over internal, experiential, and sometimes non-logical self-knowledge (Thompson et al., 2018). This highlights a potential conflict between a rationally-oriented education system and the cultivation of certain intuitive and depth-oriented dimensions of personality. Furthermore, we need reflect a potential disconnection between modern Chinese higher education curricula and traditional cultural values. The emphasis on technical skills, standardized testing, and material success metrics in advanced education might inadvertently marginalize introspective, ethical, and spiritually-oriented self-cultivation that underpins Self- *Xin* and Archetypal *Xin* . The pedagogical focus on critical rationality and external validation could distance students from the intuitive, experiential, and community-based knowledge transmission that traditionally nourished these deeper dimensions of character. This finding calls for educational reforms that integrate humanities, philosophy, and practices fostering self-awareness and ethical reflection into STEM and professional curricula, thereby bridging the gap between modern expertise and cultural wisdom.

##### 4.6.2.3 Ethnic differences

The significantly higher recognition of Self-*Xin* and Archetypal *Xin* among ethnic minorities compared to the *Han* majority is telling. It suggests that communities with potentially stronger ties to traditional beliefs, cohesive social structures, and a slower pace of modernization may provide a more conducive environment for nurturing the deep, intrinsic roots of this personality quality (Yang and Wang, 2014). For the more modernized and dispersed Han population, the very forces of modernization and social fragmentation may be associated with a weakening of the internal, foundational aspects of *Xin*.

#### 4.6.3 Understanding and reflection on non-significant results

The non-significant result regarding age indicates that the understanding and identification with *Xin* may not be a simple function of maturation or life experience in today’s China. This stability across age cohorts could imply that the core level of this personal quality is largely shaped by the broader, shared cultural atmosphere and educational milieu, which affects all generations similarly in this regard, or that its primary logic remains egocentric and relationally-based throughout adulthood.

Irrespective of the underlying causes, these observations underscore the profound influence of diverse historical and cultural contexts on the formation and development of individual inner *Xin* (Fukuyama, 2001). As Chen (2012) posited, “The process of selecting honesty or dishonesty originates from inner convictions, is shaped by external circumstances, and ultimately concludes with a choice made in response to psychological stimuli.” When individuals harbor a propensity to trust their environment, they are more prone to exhibit altruistic behaviors, motivating the development of inner *Xin* (Yu, 2012). Consequently, the cultivation of internal *Xin* necessitates not only the integration of daily practices, experiences, and insights but also the establishment of an external milieu that is fundamentally predicated on *Xin*.

#### 4.6.4 Conclusion and synthesis

In summary, the results of Study 3 do more than just describe current attitudes; they reveal the dynamic tension within the psychological structure of *Xin* as a personal quality in modern China. The high value placed on its connective, socially-integrative function exists alongside a relative neglect of its deep, archetypal core. This pattern, compounded by the influences of gender socialization, educational focus, and cultural modernization, paints a picture of a personality trait whose full and balanced development faces significant contemporary challenges.

These findings powerfully reframe the societal “crisis of dis-*Xin*” not merely as an ethical failure, but as a psychological issue rooted in the underdevelopment and disconnection of specific dimensions of a key personality quality. Therefore, fostering *Xin* requires more than moral exhortation; it calls for culturally-informed efforts aimed at nurturing the entire spectrum of this personality structure, from its deepest archetypal foundations to its integrative social expressions, creating environments where this foundational aspect of character can flourish.

## 5 Reflection and insights

While the preceding sections have delineated a range of contemporary societal issues pertaining to the erosion of *Xin* , it is equally important to acknowledge that certain aspects of this phenomenon are undergoing positive transformation. The resurgence of interest in traditional Chinese culture, catalyzed by its growing recognition and valorization, has prompted a renaissance of cultural re-engagement. This renewed focus on cultural heritage is significant because *Xin* is intrinsically woven into the fabric of culture (Wang, 2023), and bolstering cultural confidence is pivotal in revitalizing the latent *Xin* embedded in it. Although the future trajectory of cultural evolution remains unpredictable, it is imperative to recognize that *Xin* is often accompanied by its antithesis, dis-*Xin*. The evolution of *Xin* is inherently characterized by a coexistence of opportunities and challenges. The crux of the matter lies in the ability to seize pivotal moments and effectuate integration and transformation, thereby fostering a more resilient and trusting society.

Based on the empirical findings and theoretical analysis presented, we offer several critical reflections concerning the development of *Xin* as a personality trait within Chinese cultural context and its evolving significance in individual psychology.

### 5.1 *Xin* as a culturally-grounded personality construct

The global resurgence of interest in Chinese cultural paradigms necessitates a deeper understanding of its psychologically relevant constructs (Ting et al., 2019). Our research demonstrates that *Xin* can be systematically conceptualized and measured as a multifaceted personality trait, bridging philosophical discourse with empirical psychology. This represents a fundamental shift from viewing *Xin* as merely an external moral injunction to recognizing it as an internal, structural component of personality organization. Once internalized and valued, this trait can become a cornerstone for navigating psychological conflict and facilitating personal transformation.

#### 5.1.1 The dialectical nature of trait development

While exploring the archetypal and unconscious dimensions of *Xin* reveals its depth-psychological foundations, our findings equally emphasize that its development remains inextricably linked to tangible daily experiences. The authentic expression of this personality trait is realized through consistent alignment between internal states and external actions. Engagement in concrete life activities—including physical exercise (Kanaros and Bourdaniotis, 2025), dedicated learning, and mindful self-care—serves as the essential training ground for *Xin*. These practices foster a coherent and fulfilled self, demonstrating that the cultivation of profound personality traits emerges from the integrity of embodied experience.

#### 5.1.2 Systemic influences on trait manifestation

The development of *Xin* within an individual cannot be isolated from broader socio-cultural systems, the manifestation and strength of this trait are significantly shaped by cultural narratives (Mendoza, 2025), educational paradigms (Rettinger and Bertram Gallant, 2022), and familial socialization (Flores and Springer, 2021). Just as a secure therapeutic environment fosters psychological growth (Podolan, 2025), a societal system that collectively values and embodies *Xin* provides the necessary foundation for its cultivation at the individual level. This perspective highlights the essential reciprocity between personal disposition and cultural context in personality development.

## 6 Limitations and future directions

While this study strives for rigor, it is nevertheless subject to several limitations that warrant attention in future research.

First, the conceptualization of *Xin* primarily draws from classical philosophical sources, particularly the *I Ching* and Confucian classics. Future research would benefit from incorporating a wider spectrum of Chinese cultural texts and intellectual traditions to construct a more comprehensive theoretical framework.

Second, although our sampling strategy aimed for diversity, all participants were recruited from mainland China. The generalizability of findings to Chinese diaspora communities and cross-cultural applications remains to be established. Subsequent studies should expand sampling to include populations from Hong Kong, Macao, Taiwan, and overseas Chinese communities to validate the model’s cross-context stability.

Methodologically, despite employing a mixed-methods approach, potential researcher bias cannot be entirely eliminated. Future investigations could enhance objectivity through independent parallel analysis by multiple research teams, broader expert consultation, and implementation of fully standardized measurement protocols across different cultural contexts (Acar, 2024).

## 7 Conclusion

This research program has undertaken the systematic investigation of *Xin*—a cornerstone of Chinese philosophical heritage—as a measurable personality construct. Through the development and validation of a five-dimensional model and corresponding assessment instrument, we have demonstrated that *Xin* can be rigorously studied as a culturally-grounded personality trait. The documented variations in its recognition across dimensions and demographic groups underscore its nature as a dynamic individual-difference variable, deeply interwoven with socio-cultural positioning.

The evolving integration between cultural psychology and psychological personality science (Heidi, 2016), exemplified by the methodological synergy of qualitative and quantitative approaches in this study, provides fertile ground for advancing indigenous psychology. This research establishes an empirical foundation for future studies to explore the developmental pathways, behavioral correlates, and well-being implications of *Xin* as a core aspect of personality functioning in Chinese contexts. By bridging cultural philosophy with contemporary personality psychology, this work contributes to developing more culturally-informed models of human individuality and psychological functioning.

## Data Availability

The original contributions presented in the study are included in the article/Supplementary material, further inquiries can be directed to the corresponding authors.
