# Supplementary material for: An exploration of the structure and understanding of Xin (“信”) in Chinese culture: the development of a theoretical model and questionnaire instruments
Source: Front Psychol. 2026 Feb 27;17:1762903. doi: 10.3389/fpsyg.2026.1762903 (PMC12983231; doi:10.3389/fpsyg.2026.1762903)
Supplement: Supplementary file 1 [file Supplementary_file_1.docx]

# Appendix A

# Initial Items for Questionnaire on Xin (“信”) in Chinese Culture and Its Construct

中国文化中的“信”及其结构问卷（52题）

**指导语：**您好！这是一份关于您是如何理解中国文化中“信”的内涵的问卷，我们将根据您的回答更好地验证中国文化中的“信”及其结构，并发掘“信”在我们日常生活及心灵深处的重要作用。因此，您的参与对我们来说非常重要！答案没有好坏、对错之分，请您根据您的真实观点作答。

我们郑重承诺：您的信息将会被匿名处理且仅用于本研究使用，请放心填写！

- 性别
- 年龄
- 学历水平
- 职业行业
- 所在地区

| 题号 | 请根据您的真实想法给下列问题打分：您同意以下观点吗？1-5分。  （1:非常不同意；2:比较不同意；3:不知道；4:比较同意；5:非常同意） |
| --- | --- |
| 1 | 信是个体和其他人或事物产生的连接。 |
| 2 | 信会表现为一种相互制约、相互制衡的约定。 |
| 3 | 可以通过签合同、约定、抵押、交换的方式加强人与人之间的信。 |
| 4 | 信看不见也摸不着，但我们能感受和体验到。 |
| 5 | 信是我们对未来抱有希望的基础。 |
| 6 | 一个人的内心真正有信，才能和其他人、社会或自然和谐相处。 |
| 7 | 信是世界上所有事物存在和发展的基础。 |
| 8 | 信是一个人与所有事物产生关系的基础。 |
| 9 | 信是一个人活在世界上最根本的依靠。 |
| 10 | 信是一种真实的体现，代表了内心的真与诚。 |
| 11 | 信会使一个人变得有力量。 |
| 12 | 人和人交往，最重要的就是讲信用。 |
| 13 | 不带个人偏见、无差别的信才是真正的信。 |
| 14 | 信可以表现为一个人是可靠的。 |
| 15 | 信是做了承诺就要履行它。 |
| 16 | 因为不信而导致不稳定需要依靠其他事物来巩固。 |
| 17 | 信能够促使社会稳定发展。 |
| 18 | 信是一种道德品性。 |
| 19 | 信能够推动对新事物的探索和发现。 |
| 20 | 信会改变人或事物之间的关系。 |
| 21 | 信能够推动人的心理的发展。 |
| 22 | 信出现在人类社会，是人类历史发展和进步的结果。 |
| 23 | 信是人与天地之间有所感应的必备条件。 |
| 24 | 孩子和妈妈之间的相互信赖就是一种信的体现。 |
| 25 | 信可以平衡、调节不对等的事物。 |
| 26 | 信在所有事物中是普遍存在的。 |
| 27 | 信可以为一个人的成长提供空间。 |
| 28 | 信可以通过提供能量来充实并滋养人的内心。 |
| 29 | 信像天理、法则一样是毋庸置疑的。 |
| 30 | 信会以信息的形式存在于万物之中。 |
| 31 | 信可以表现为太阳东升西落，春夏秋冬的轮转。 |
| 32 | 世间万物都在变化，但信会一直存在。 |
| 33 | 一个人内心中的自我的诞生源自信的出现。 |
| 34 | 放下自己的偏见才能建立一个人内心中的信。 |
| 35 | 每个人心中都有自己不同的信。 |
| 36 | 真正的信是永远都不会消失的，只是人不一定随时看得到。 |
| 37 | 信会激发一个人的潜能。 |
| 38 | 信能够依靠个体内在的力量使个体的内心发展完善。 |
| 39 | 信代表了事物真实的样子，没有任何修饰或伪装。 |
| 40 | 信是时间和历史的积淀。 |
| 41 | 信是一种敬畏之心。 |
| 42 | 信可以表现为对神灵的敬仰和尊重 |
| 43 | 信会使一个人的内心发生改变。 |
| 44 | 信是一个人智慧的开端。 |
| 45 | 信是超出一个人意识范围的事物存在的依据。 |
| 46 | 信会对一个人的行为起到指导或者驱动的作用。 |
| 47 | 如果一个人自己不信，那TA展现给别人的也是虚假的信。 |
| 48 | 信是一种由衷而发的态度。 |
| 49 | 发自内心的信会自然而然的表现在一个人的行为中。 |
| 50 | 信会改变事情的发展和结果。 |
| 51 | 一个人真正的信是不会被别人左右的。 |
| 52 | 真正的信是永远都不会消失的，只是不一定随时可以看到。 |

**Questionnaire on *Xin* (“信”) in Chinese Culture and Its Construct (52 Items)**

**Instructions:** This questionnaire is designed to explore your understanding of the connotation of Xin (“信”) within Chinese cultural contexts. Your responses will contribute to validating the construct of Xin in Chinese culture and explore its significant role in daily life and inner spirituality. Thus, your participation is highly valuable to us! There are no correct or incorrect answers, please respond based on your genuine perspectives. We solemnly commit that your information will be anonymized and used solely for this research. Please feel assured in providing your responses!

- Gender
- Age
- Educational Level
- Occupation/Industry
- Geographic Region

| Item | Please rate the following statements based on your genuine opinion: To what extent do you agree with each of the following statements? Use a scale from 1 to 5. (1: Strongly disagree; 2: Disagree; 3: Neutral; 4: Agree; 5: Strongly agree) |
| --- | --- |
| 1 | *Xin* represents a connection between a person and other people or things. |
| 2 | *Xin* can manifest as a mutually restraining and balancing agreement. |
| 3 | *Xin* between people can be strengthened through contracts, agreements, collateral, or exchanges. |
| 4 | *Xin* is intangible and invisible, but it can be felt and experienced. |
| 5 | *Xin* serves as the foundation for our hope in the future. |
| 6 | Only when one truly possesses *Xin* internally can they harmoniously coexist with others, society, or nature. |
| 7 | *Xin* is the foundation for the existence and development of all things in the world. |
| 8 | Xin forms the foundation for an individual's relationship with all things. |
| 9 | *Xin* is the most fundamental reliance for an individual’s existence in the world. |
| 10 | *Xin* is a manifestation of authenticity, representing inner truth and sincerity. |
| 11 | *Xin* empowers an individual with strength. |
| 12 | The most important aspect of interpersonal interaction is keeping one’s *Xin*. |
| 13 | Genuine *Xin* is impartial and unbiased, without discrimination. |
| 14 | *Xin* can be reflected in a person’s reliability. |
| 15 | *Xin* means fulfilling a promise once it is made. |
| 16 | Instability caused by a lack of *Xin* requires reinforcement through other means. |
| 17 | *Xin* can promote the stable development of society. |
| 18 | *Xin* is a moral character trait. |
| 19 | *Xin* can drive the exploration and discovery of new things. |
| 20 | *Xin* can transform the relationships between people and things. |
| 21 | *Xin* can facilitate psychological development in individuals. |
| 22 | The emergence of *Xin* in human society is a result of historical development and progress. |
| 23 | *Xin* is a prerequisite for the resonance between humans and the natural world. |
| 24 | The mutual trust between a child and their mother is one manifestation of *Xin*. |
| 25 | *Xin* can balance and regulate unequal or asymmetrical relationships. |
| 26 | *Xin* exists universally across all things. |
| 27 | *Xin* can provide space for an individual’s growth. |
| 28 | *Xin* can enrich and nurture a person’s inner self by providing energy. |
| 29 | Like the cosmic order, *Xin* is inherently indubitable. |
| 30 | *Xin* exists in all things in the form of information. |
| 31 | The natural order，such as the sunrise and sunset, and the succession of seasons exemplifies *Xin*. |
| 32 | All things in the world are in flux, but *Xin* endures. |
| 33 | The emergence of the self within an individual originates from the presence of *Xin*. |
| 34 | To cultivate inner *Xin*, one must first relinquish personal prejudice. |
| 35 | Each person holds their own distinct understanding of *Xin*. |
| 36 | *Xin* endures permanently, though it may not always be visible to human perception |
| 37 | *Xin* can stimulate an individual’s potential. |
| 38 | Relying on inner strength, *Xin* enables the development and refinement of an individual’s inner self. |
| 39 | *Xin* represents the true nature of things, without embellishment or disguise. |
| 40 | *Xin* is an accumulation over time and history. |
| 41 | *Xin* is a form of reverence. |
| 42 | *Xin* can be manifested as reverence and respect for deities. |
| 43 | *Xin* can bring about an inner transformation in a person. |
| 44 | *Xin* marks the beginning of a person's wisdom. |
| 45 | *Xin* serves as the foundation for the existence of things beyond an individual's conscious awareness. |
| 46 | *Xin* can guide and motivate an individual's behavior. |
| 47 | If a person dis-*Xin* internally, any *Xin* they display to others is false. |
| 48 | *Xin* is an attitude that arises genuinely from within. |
| 49 | *Xin* that comes from the heart naturally manifests in a person’s behavior. |
| 50 | *Xin* can influence the course and outcome of events. |
| 51 | A person’s genuine *Xin* cannot be swayed by others. |
| 52 | True *Xin* never disappears, though it may not always be visible. |
