# Supplementary material for: An exploration of the structure and understanding of Xin (“信”) in Chinese culture: the development of a theoretical model and questionnaire instruments
Source: Front Psychol. 2026 Feb 27;17:1762903. doi: 10.3389/fpsyg.2026.1762903 (PMC12983231; doi:10.3389/fpsyg.2026.1762903)
Supplement: Supplementary file 2 [file Supplementary_file_2.docx]

**Appendix B**

**Questionnaire on Xin (“信”) in Chinese Culture and Its Construct**

中国文化中的“信”及其结构问卷（42题）

**指导语：**您好！这是一份关于您是如何理解中国文化中“信”的内涵的问卷，我们将根据您的回答更好地验证中国文化中的“信”及其结构，并发掘“信”在我们日常生活及心灵深处的重要作用。因此，您的参与对我们来说非常重要！答案没有好坏、对错之分，请您根据您的真实观点作答。

我们郑重承诺：您的信息将会被匿名处理且仅用于本研究使用，请放心填写！

- 性别
- 年龄
- 民族
- 所在城市
- 学历水平
- 职业

| 题号 | 请根据您的真实想法给下列问题打分：您同意以下观点吗？1-5分。 （1:非常不同意；2:比较不同意；3:不知道；4:比较同意；5:非常同意） |
| --- | --- |
| 1 | 信是个体和其他人或事物产生的连接。 |
| 2 | 信会表现为一种相互制约、相互制衡的约定。 |
| 3 | 可以通过签合同、约定、抵押、交换的方式加强人与人之间的信。 |
| 4 | 信看不见也摸不着，但我们能感受和体验到。 |
| 5 | 信是我们对未来抱有希望的基础。 |
| 6 | 一个人的内心真正有信，才能和其他人、社会或自然和谐相处。 |
| 7 | 信是世界上所有事物存在和发展的基础。 |
| 8 | 信是一个人活在世界上最根本的依靠。 |
| 9 | 信是一种真实的体现，代表了内心的真与诚。 |
| 10 | 信会使一个人变得有力量。 |
| 11 | 人和人交往，最重要的就是讲信用。 |
| 12 | 不带个人偏见、无差别的信才是真正的信。 |
| 13 | 信可以表现为一个人是可靠的。 |
| 14 | 信是做了承诺就要履行它。 |
| 15 | 因为不信而导致不稳定需要依靠其他事物来巩固。 |
| 16 | 信能够促使社会稳定发展。 |
| 17 | 信是一种道德品性。 |
| 18 | 信能够推动对新事物的探索和发现。 |
| 19 | 信能够推动人的心理的发展。 |
| 20 | 信出现在人类社会，是人类历史发展和进步的结果。 |
| 21 | 信可以平衡、调节不对等的事物。 |
| 22 | 信在所有事物中是普遍存在的。 |
| 23 | 信可以为一个人的成长提供空间。 |
| 24 | 信可以通过提供能量来充实并滋养人的内心。 |
| 25 | 信会以信息的形式存在于万物之中。 |
| 26 | 世间万物都在变化，但信会一直存在。 |
| 27 | 一个人内心中的自我的诞生源于信的出现。 |
| 28 | 每个人心中都有自己不同的信。 |
| 29 | 信会激发一个人的潜能。 |
| 30 | 信能够依靠个体内在的力量使个体的内心发展完善。 |
| 31 | 信代表了事物真实的样子，没有任何修饰或伪装。 |
| 32 | 信是时间和历史的积淀。 |
| 33 | 信是一种敬畏之心。 |
| 34 | 信会使一个人的内心发生改变。 |
| 35 | 信是超出一个人意识范围的事物存在的依据。 |
| 36 | 信会对一个人的行为起到指导或者驱动的作用。 |
| 37 | 如果一个人自己不信，那TA展现给别人的也是虚假的信。 |
| 38 | 信是一种由衷而发的态度。 |
| 39 | 发自内心的信会自然而然的表现在一个人的行为中。 |
| 40 | 信会改变事情的发展和结果。 |
| 41 | 一个人真正的信是不会被别人左右的。 |
| 42 | 真正的信是永远都不会消失的，只是不一定随时可以看到。 |

**Questionnaire on Xin (“信”) in Chinese Culture and Its Construct (42 items)**

**Instructions:** This questionnaire is designed to explore your understanding of the connotation of Xin (“信”) within Chinese cultural contexts. Your responses will contribute to validating the construct of Xin in Chinese culture and explore its significant role in daily life and inner spirituality. Thus, your participation is highly valuable to us! There are no correct or incorrect answers, please respond based on your genuine perspectives.

We solemnly commit that your information will be anonymized and used solely for this research. Please feel assured in providing your responses!

- Gender
- Age
- Ethnicity
- City
- Educational Level
- Career

| Item | Please rate the following statements based on your genuine opinion: To what extent do you agree with each of the following statements? Use a scale from 1 to 5. (1: Strongly disagree; 2: Disagree; 3: Neutral; 4: Agree; 5: Strongly agree) |
| --- | --- |
| 1 | *Xin* represents a connection between a person and other people or things. |
| 2 | *Xin* can manifest as a mutually restraining and balancing agreement |
| 3 | *Xin* between people can be strengthened through contracts, agreements, collateral, or exchanges. |
| 4 | *Xin* is intangible and invisible, but it can be felt and experienced. |
| 5 | *Xin* serves as the foundation for our hope in the future. |
| 6 | Only when one truly possesses *Xin* internally can they harmoniously coexist with others, society, or nature. |
| 7 | *Xin* is the foundation for the existence and development of all things in the world. |
| 8 | *Xin* is the most fundamental reliance for an individual’s existence in the world. |
| 9 | *Xin* is a manifestation of authenticity, representing inner truth and sincerity. |
| 10 | *Xin* empowers an individual with strength. |
| 11 | The most important aspect of interpersonal interaction is keeping one’s word. |
| 12 | Genuine *Xin* is impartial and unbiased, without discrimination. |
| 13 | *Xin* can be reflected in a person’s reliability. |
| 14 | *Xin* means fulfilling a promise once it is made. |
| 15 | Instability caused by a lack of *Xin* requires reinforcement through other means. |
| 16 | *Xin* can promote the stable development of society. |
| 17 | *Xin* is a moral character trait. |
| 18 | *Xin* can facilitate the exploration and discovery of new things. |
| 19 | *Xin* can facilitate psychological development in individuals. |
| 20 | The emergence of *Xin* in human society is a result of historical development and progress. |
| 21 | *Xin* can balance and regulate disparities and inequalities. |
| 22 | *Xin* exists universally across all things. |
| 23 | *Xin* can provide space for an individual’s growth. |
| 24 | *Xin* can enrich and nurture a person’s inner self by providing energy. |
| 25 | *Xin* exists in all things in the form of information. |
| 26 | All things in the world are in flux, but *Xin* endures. |
| 27 | The emergence of the inner self originates from the presence of *Xin*. |
| 28 | Each person holds their own distinct understanding of *Xin*. |
| 29 | *Xin* can stimulate an individual’s potential. |
| 30 | Relying on inner strength, *Xin* enables the development and refinement of an individual’s inner self. |
| 31 | *Xin* represents the true nature of things, free from embellishment or disguise. |
| 32 | *Xin* is an accumulation shaped by time and history. |
| 33 | *Xin* embodies a sense of reverence. |
| 34 | *Xin* can bring about inner transformation in person. |
| 35 | *Xin* serves as the basis for the existence of things beyond an individual’s conscious awareness. |
| 36 | *Xin* can guide or motivate a person’s behavior. |
| 37 | If a person has no genuine *Xin* internally, any *Xin* they display to others is false. |
| 38 | *Xin* is an attitude that arises sincerely from within. |
| 39 | *Xin* that comes from the heart naturally manifests in a person’s behavior. |
| 40 | *Xin* can influence the course and outcome of events. |
| 41 | A person’s genuine *Xin* cannot be swayed by others. |
| 42 | True *Xin* never disappears, though it may not always be visible. |
